# Wolf’s isotopic response of eczema after herpes zoster infection: case report and literature review

**DOI:** 10.3389/fmed.2025.1697012

**Published:** 2026-01-05

**Authors:** Yirui Zhao, Min Zhang, Xuanlin Chen, Shuping Guo, Hongye Liu

**Affiliations:** 1 The First Clinical Medical College, Shanxi Medical University, Taiyuan, Shanxi, China; 2 Department of Dermatology, First Hospital of Shanxi Medical University, Taiyuan, Shanxi, China

**Keywords:** herpes zoster, eczema, Wolf’s isotopic response, neuropeptides, Th2-type immune responses

## Abstract

Wolf’s post-herpetic isotopic response (PHIR) refers to the development of new cutaneous conditions, such as tumors, infections, or immune-mediated diseases, at the site of a previously healed herpetic eruption. The exact pathogenesis of Wolf’s PHIR remains incompletely understood. Four hypotheses have been proposed regarding the mechanism of postherpetic inflammatory isomorphic reactions: viral, immunological, vascular, and neurological. In this case, we show that a patient with eczema developed it at the original site following the resolution of the herpes zoster infection. After 2 weeks of systemic oral glucocorticoid treatment, the skin lesions improved significantly, and there was no recurrence during the 3-month follow-up.

## Introduction

The concept of Wolf’s isotopic response was introduced by Wolf et al. in 1995 and is defined as the development of a secondary disease with distinct characteristics at the same anatomical site following the resolution of the primary condition (1). This report presents a rare case of eczema arising as an isotopic response to herpes zoster infection.

## Case reports

**Figure 1:**
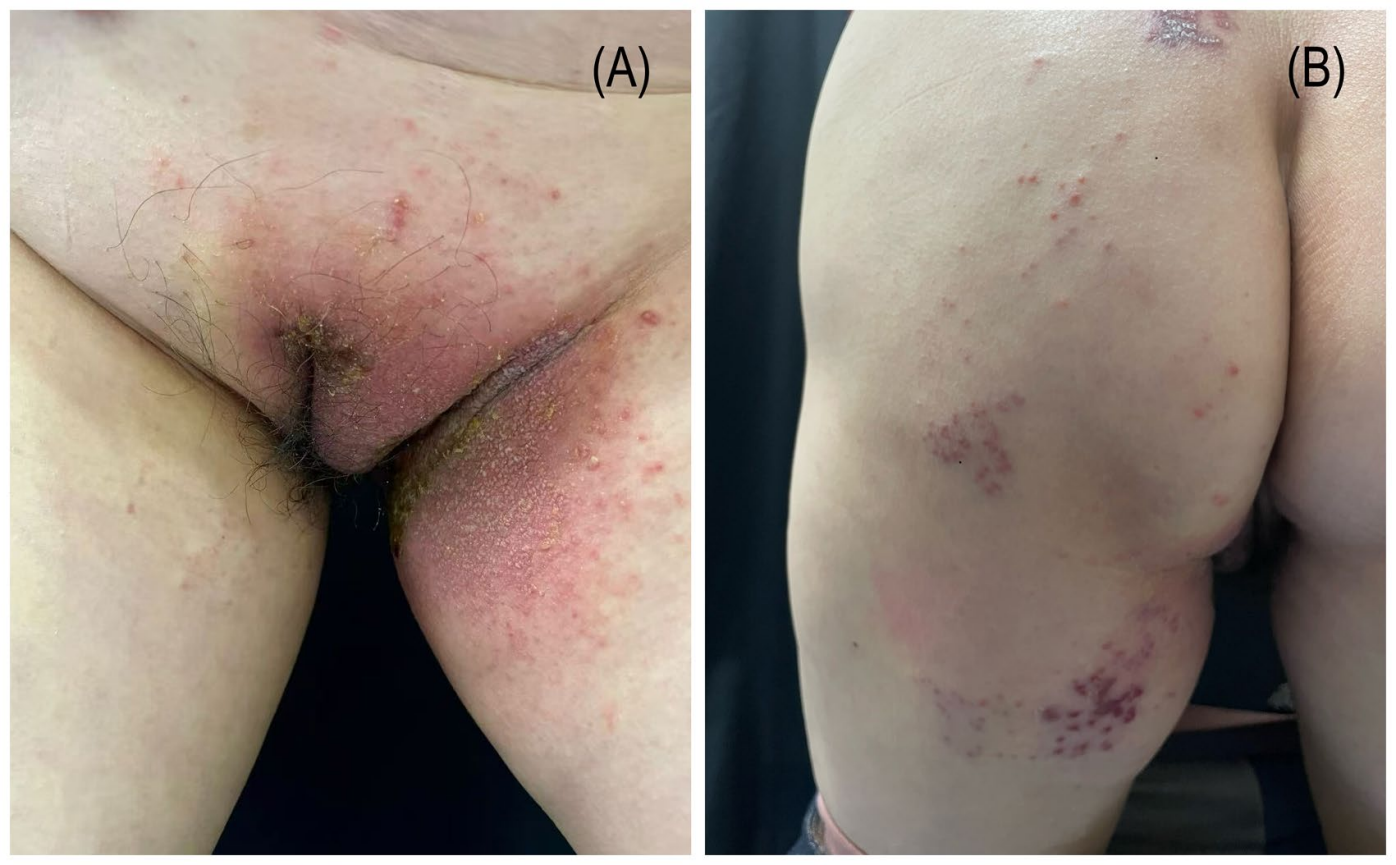
Erythema, papules, exudation, and crusting observed on the left perineum **(A)** and left lower **(B)** extremity are localized to the previously healed site of a herpes zoster lesion.

**Figure 2:**
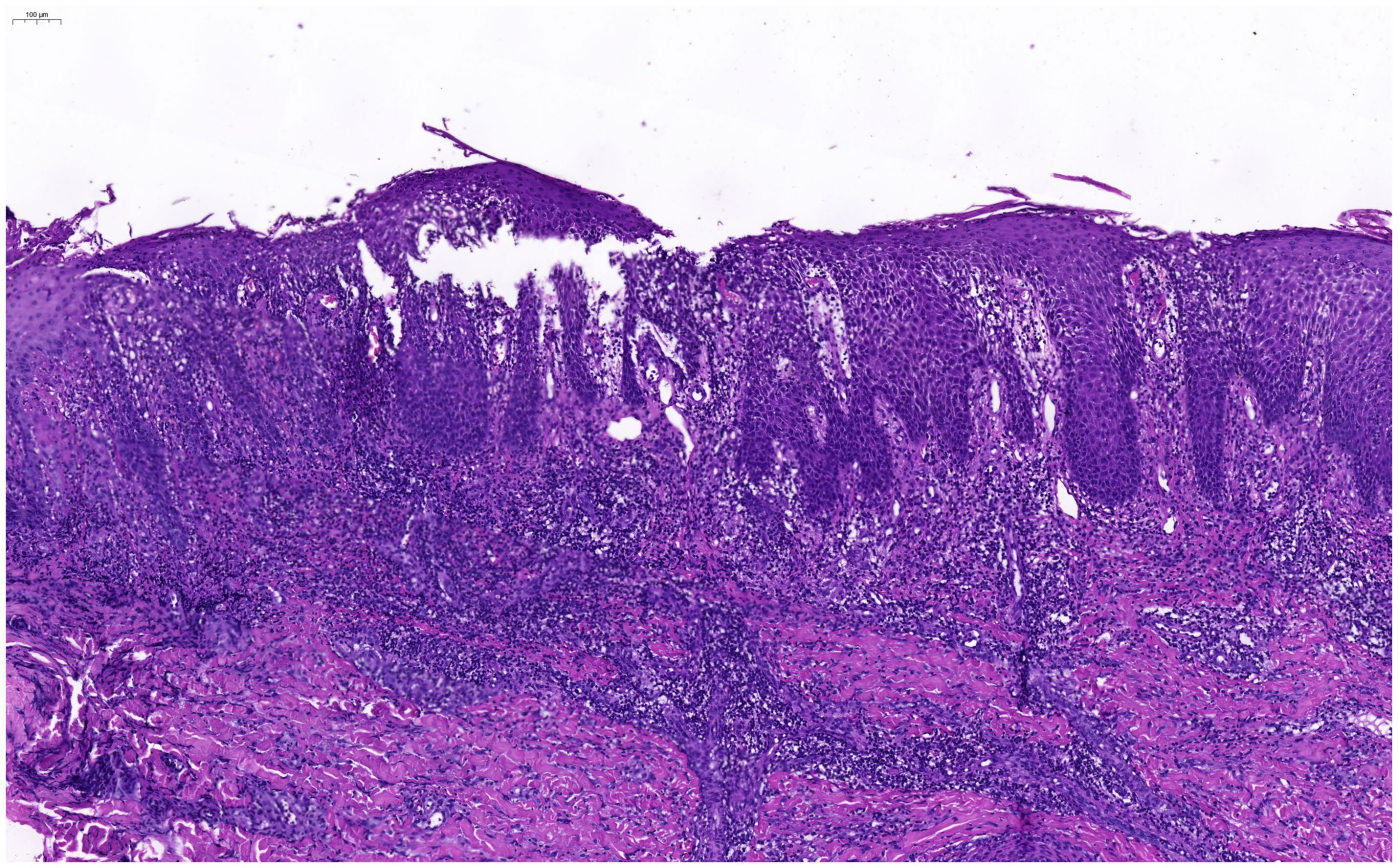
Pathological findings indicate the presence of spongiotic changes consistent with spongiotic dermatitis (H&E200×).

A 54-year-old woman presented to our department with a 5-day history of rash and pruritus on the left perineum and left lower limb (for a detailed timeline, see Table 1). She had no personal or family history of atopic diseases. Forty days earlier, she had been diagnosed with herpes zoster affecting the left perineum and left lower limb. Following antiviral treatment, the skin lesions healed. On physical examination, erythematous plaques and papules were observed at the previous site of herpes zoster infection, accompanied by exudation and crusting (Figure 1). Histopathological examination revealed spongiotic dermatitis (Figure 2). Based on the clinical presentation and histopathological findings, a diagnosis of non-specific eczematous dermatitis was established. After 14 days of oral administration of prednisone acetate tablets (30 mg per day) and topical application of fluticasone propionate cream (twice a day), the patient’s rash improved significantly. Three months later, the patient’s eczema did not recur.

**Table 1:** Disease diagnosis and treatment progress.

Time point	Clinical and intervention measures	Clinical results and findings
40 days ago	Diagnosed with herpes zoster in the left perineum/lower limb, given antiviral treatment.	Herpes zoster lesions healed.
5 days ago	New pruritic rash at the original site.	Eczema occurred.
Visit Day (day 0)	Found erythema, papules, and exudation; underwent pathology.	Diagnosed as eczema clinically/pathologically.
Days 1–14	Treated with oral prednisone acetate (30 mg/d) and topical fluticasone propionate cream (twice daily).	Rash improved notably.
Days 15 to 3 months	Entered clinical follow-up phase.	Skin lesions resolved completely, no recurrence.

## Discussion

Wolf’s post-herpetic isotopic response (PHIR) refers to the development of new cutaneous conditions, such as tumors, infections, or immune-mediated diseases, at the site of a previously healed herpetic eruption (2). The exact pathogenesis of Wolf’s PHIR remains incompletely understood. Four hypotheses have been proposed regarding the mechanism of postherpetic inflammatory isomorphic reactions: viral, immunological, vascular, and neurological.

The patient had no history of specific contact with or eczema. Clinical and histopathological findings confirmed eczematous changes, consistent with an isotopic response to prior herpes zoster infection at the same site. We hypothesize that the viral infection may damage A-*δ* and C nerve fibers in the mid-to-deep dermis, leading to reduced fiber density and the release of neuropeptides from local neural tissues. These neuropeptides include substance P, vasoactive intestinal peptide (VIP), bradykinin, serotonin, calcitonin gene-related peptide (CGRP), and *α*-melanocyte-stimulating hormone (α-MSH) (3). Studies indicate that substance P can induce mast cells and keratinocytes to release pruritogens via neurokinin 1 receptor activation, potentially activating Mas-related G protein-coupled receptor X2 (MRGPRX2) to trigger mast cell degranulation and pruritus transmission (4). CGRP enhances Th2-mediated immune responses (5). Type 2 inflammatory responses impair skin barrier function by inhibiting filaggrin gene expression and disrupting its reticular structure. The development of pruritus, compromised skin barrier integrity, and enhanced Th2-type immune responses collectively contribute to the onset of eczema.

Currently, cases of eczema secondary to herpes zoster infection are rarely documented in the literature. We reviewed four confirmed cases based on the existing reports. The time intervals between the onset of herpes zoster and the subsequent development of eczema were relatively short, as shown in the data (Table 2). These cases have a high proportion of females in terms of demographics and a wide age range; in terms of time, the delayed onset is mainly at 4 weeks and 6 weeks. The recurrence of erythema, papules, and pustules at the previously healed site of herpes zoster was histopathologically confirmed as eczema. However, such presentations are often misinterpreted as a recurrence of herpes zoster, potentially leading to unnecessary antiviral therapy.

**Table 2:** Characteristics of reported cases with eczema after herpes zoster infection.

Source	Age/sex	Delay	Location	Relapse
Our case	54/F	4 weeks	Left perineum, groin, and buttocks	No
Zijia et al. (2021) (6)	55/F	6 weeks	Lumbar/abdominal	No
Yijun et al. (2018) (7)	74/F	6 weeks	Left groin	No
Ling et al. (2016) (8)	25/M	4 weeks	Right thoracodorsal region	No

## Conclusion

However, this study has several limitations. The diagnosis primarily relies on histopathological examination and clinical presentation, and the causal relationship with the previous herpes infection remains inferential. As a single case report, the generalizability of the conclusions is limited. Although the proposed neuropeptide-mediated immune activation mechanism is supported by the existing literature, it lacks molecular-level validation in this particular case. Therefore, the precise molecular mechanisms underlying this rare phenomenon, particularly the pathway linking neuronal injury, neuropeptide release, and cutaneous inflammation, warrant further elucidation through more extensive basic and clinical research.

## Data Availability

The original contributions presented in the study are included in the article/supplementary material, further inquiries can be directed to the corresponding authors.
